# Systematic scoping review of automated systems for the surveillance of healthcare-associated bloodstream infections related to intravascular catheters

**DOI:** 10.1186/s13756-024-01380-x

**Published:** 2024-02-28

**Authors:** Nasim Lotfinejad, Jean-Marie Januel, Sarah Tschudin-Sutter, Peter W Schreiber, Bruno Grandbastien, Lauro Damonti, Elia Lo Priore, Alexandra Scherrer, Stephan Harbarth, Gaud Catho, Niccolò Buetti, Swissnoso Group

**Affiliations:** 1 Infection Control Program and WHO Collaborating Centre, Geneva University Hospitals and Faculty of Medicine, Geneva, Switzerland; 2 Division of Infectious Diseases & Hospital Epidemiology, University Hospital Basel and University of Basel, Basel, Switzerland; 3 Department of Infectious Diseases and Hospital Epidemiology, University Hospital Zurich and University of Zurich, Zurich, Switzerland; 4 Infection Prevention and Control Unit, Service of Infectious Disease, Lausanne University Hospital, Lausanne, Switzerland; 5 Department of Infectious Diseases, Bern University Hospital, University of Bern, Bern, Switzerland; 6 Department of Infectious Diseases and Hospital Epidemiology, EOC Regional Hospital of Lugano, Lugano, Switzerland; 7 National Center for Infection Control, Swissnoso, Bern, Switzerland; 8 Division of Infectious Diseases, Central Institute, Valais Hospital, Sion, Switzerland; 9 Université Paris-Cité, INSERM, IAME UMR 1137 , Paris, 75018 France

**Keywords:** CLABSI, CRBSI, Automated monitoring, Algorithm, Surveillance, Healthcare associated infections, Automation

## Abstract

**Introduction:**

Intravascular catheters are crucial devices in medical practice that increase the risk of healthcare-associated infections (HAIs), and related health-economic adverse outcomes. This scoping review aims to provide a comprehensive overview of published automated algorithms for surveillance of catheter-related bloodstream infections (CRBSI) and central line-associated bloodstream infections (CLABSI).

**Methods:**

We performed a scoping review based on a systematic search of the literature in PubMed and EMBASE from 1 January 2000 to 31 December 2021. Studies were included if they evaluated predictive performance of automated surveillance algorithms for CLABSI/CRBSI detection and used manually collected surveillance data as reference. We assessed the design of the automated systems, including the definitions used to develop algorithms (CLABSI *versus* CRBSI), the datasets and denominators used, and the algorithms evaluated in each of the studies.

**Results:**

We screened 586 studies based on title and abstract, and 99 were assessed based on full text. Nine studies were included in the scoping review. Most studies were monocentric (*n* = 5), and they identified CLABSI (*n* = 7) as an outcome. The majority of the studies used administrative and microbiological data (*n* = 9) and five studies included the presence of a vascular central line in their automated system. Six studies explained the denominator they selected, five of which chose central line-days. The most common rules and steps used in the algorithms were categorized as hospital-acquired rules, infection rules (infection *versus* contamination), deduplication, episode grouping, secondary BSI rules (secondary *versus* primary BSI), and catheter-associated rules.

**Conclusion:**

The automated surveillance systems that we identified were heterogeneous in terms of definitions, datasets and denominators used, with a combination of rules in each algorithm. Further guidelines and studies are needed to develop and implement algorithms to detect CLABSI/CRBSI, with standardized definitions, appropriate data sources and suitable denominators.

**Supplementary Information:**

The online version contains supplementary material available at 10.1186/s13756-024-01380-x.

## Background

Intravascular catheters (IVC) are essential devices in medical practice; however, they increase the risk of healthcare-associated infections (HAI). HAI are among the most common adverse events in healthcare settings with a mean prevalence of 6.5% in Europe [1, 2]. Central-line associated bloodstream infections (CLABSI) contribute significantly, accounting for 14.2% of all HAIs [3]. In European intensive care units (ICU), catheter-related bloodstream infection (CRBSI) represents 36.5% of acquired bloodstream infections [4]. CLABSI and CRBSI are preventable HAI, which result in increases in mortality rates, duration of hospitalization and healthcare expenditure [5–10].

Surveillance activities are deemed crucial to reduce HAI as they provide necessary information to identify problems and priorities [1]. Surveillance of bloodstream infections (BSI) related to IVC allows to quantify the burden of disease and to assess the effectiveness of interventions to prevent these infections. With this regards, the Center for Disease Control and Preventions (CDC) National Healthcare Safety Network (NHSN) has proposed to use the CLABSI definition criteria for surveillance purposes while including only central venous catheters, whereas CRBSI is a clinical definition mostly used for research investigations or clinical practice [11, 12].

Evidence suggests that automated algorithms can improve the efficiency of CLABSI/CRBSI surveillance compared to conventional “manual” surveillance, which is time consuming and resource intensive [13–15]. When designing an automated surveillance system, different points should be considered, such as definitions of HAI, data sources, algorithm development and validation against the best reference standard [14]. In our recent publication, we evaluated the predictive performance of automated algorithms for CLABSI/CRBSI detection, and we found that the performance of automated algorithms for detection of intravascular catheter infections in comparison to manual surveillance seems encouraging. In this scoping review, we aimed to provide a comprehensive overview of the relevant algorithms reported in the literature for automated surveillance of CLABSI/CRBSI surveillance.

## Methods

### Overview

This study was designed as a systematic scoping review following the Preferred Items for Systematic Reviews and Meta-Analysis guidelines extension for Scoping Reviews (PRISMA-ScR) [16]. We performed a scoping review because this type of review is appropriate when knowledge on a specific topic has not been comprehensively reviewed, as it is the case with automated algorithms for the detection of CLABSI/CRBSI in hospitalized patients. This study was registered within the PROSPERO international prospective register of systematic reviews (CRD42022299641) on January 21, 2022 [17].

### Eligibility criteria

We limited our search to studies published between January 2000 and December 2021. Eligible study designs were observational (e.g., case–control, case series, and cross-sectional studies), experimental (e.g., randomized control trials), and quasi-experimental (e.g., controlled before and after studies, interrupted time series) studies. We included studies reporting fully automated surveillance or semiautomated surveillance (including a manual determination part) of CLABSI/CRBSI. For the sake of simplicity, we opted to use the term “automated” instead of “automated *and* semiautomated” throughout the manuscript. Studies were excluded if they lacked direct relevance to automated surveillance or did not address CLABSI/CRBSI.

### Information sources

We systematically searched two electronic databases, PubMed and EMBASE, for relevant articles published between 1 January 2000 and 31 December 2021. The search was limited to articles published in English. We searched for studies that reported on automated surveillance of CLABSI/CRBSI. We performed two different searches. A search for studies that reported on the predictive performance of automated algorithms for the detection of any type of HAI (to increase the sensitivity of the search strategy) and a search for studies on the detection of IVC infections specifically (to increase the specificity of the search strategy). The records from the two searches were merged, and duplicates were removed using the EndNote program (Thomson Reuters, NY, USA).

### Search

The search for studies reporting on surveillance outcomes was performed in PubMed and EMBASE. Briefly, we included terms related to intravascular catheters, BSI or CRBSI/CLABSI, automation and surveillance. Search strategy details are illustrated in the supplementary material.

### Selection of sources of evidence

Two investigators (N.L. and J.M.J.) screened titles and abstracts and examined the full text of original articles selected for study inclusion independently and in duplicate. We resolved disagreements on study selection and data extraction by consensus and discussion with other authors if needed.

### Data charting process

Data from studies retrieved through the systematic search were extracted using Microsoft Excel. A data-charting form was jointly developed by two reviewers to determine which variables to extract. Any disagreements were resolved through discussion between the two reviewers or further adjudication by a third reviewer.

### Data items

We abstracted data on article characteristics (e.g., publication year, country of study) and on surveillance system characteristics including definitions of CLABSI/CRBSI, datasets used for the numerators and denominators, and algorithm rules. When multiple algorithms (i.e., algorithms with different definitions for identifying intravascular catheters infections) were evaluated in a single study, we defined each individual rule or combination of rules as a single observation in our study. The total number of algorithms evaluated was therefore higher than the number of included studies.

### Synthesis of results

Study characteristics were tabulated and narratively summarized. Data on definitions of CLABSI/CRBSI were summarized in the text. Data on datasets used for numerators and denominators were tabulated and narratively summarized. Algorithm rules were grouped by rule categories as ‘hospital acquisition’, ‘infection’, ‘duplication’, ‘secondary BSI’, and ‘catheter associated’. Quantitative meta-analysis of the predictive performance of these algorithms was performed, and published elsewhere [18].

## Results

### Selection of sources of evidence

**Fig. 1:**
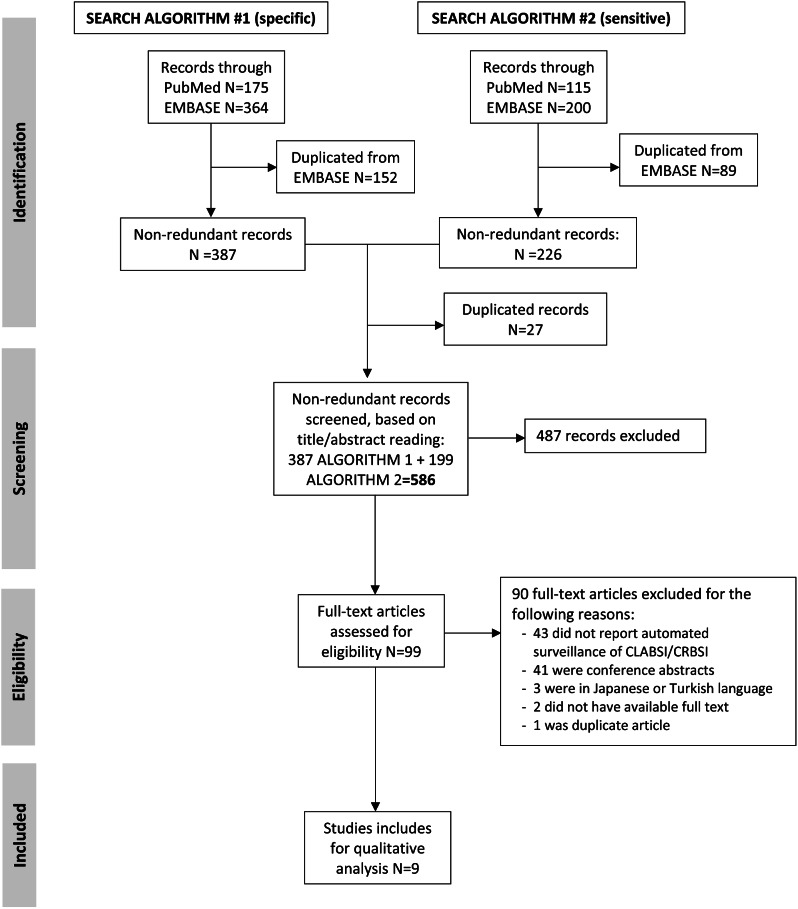
Study flow-chart

After duplicates were removed, we identified 586 non-redundant study records (Fig. 1). Based on the title and abstract screening, 487 records were excluded, with 99 full text articles to be retrieved and assessed for eligibility. Of these, 90 were excluded: 48% (*n* = 43) of studies did not report automated surveillance data for CLABSI/CRBSI.

### Characteristics of individual sources of evidence

The characteristics of the nine included studies are presented in Table 1. Most studies (*n* = 6) were conducted in the United States. Five of the studies included in the scoping review were monocentric and the remaining four were multicentric. All studies were observational and mostly focused on central venous catheters (CVC).Two studies used a surveillance based on culture-positive catheter tips; none of them specified which type of peripheral catheters they used [19, 20]. A total of 46 different automated algorithms or study populations were identified.

**Table 1:** Study characteristics

Study	Setting	Type of ward	Location	Study period	Automated vs. semiautomated	Study population sample size	Catheter types included	Outcome	Patient-days or length of stay (average)	Age of the study population
Trick et al. [21]	2 hospitals	All wards excepted neonate and pediatric wards	US, Chicago	September 1st, 2001 to February 28th, 2002	Fully- and semi-automated (including manual CVC determination)	99 patients (104 positive blood culture) in hospital 1, and 28 patients (31 positive blood culture) in hospital 2	CVC	CLABSI	NA	Median age was 52 years-old for patients hospitalized in hospital 1, and 60 years-old for patients in hospital 2
Bellini et al. [19]	1 hospital	All types	Switzerland, Lausanne	3-years period	Fully automated	669 positive blood culture	Unspecified intravascular catheters	CRBSI	NA	NA
Woeltje et al. [22]	1 hospital	6 ICU	US, Missouri	July 1st, 2005 to December 31, 2006	Fully automated	540 patients (694 positive blood culture)	CVC	CLABSI	NA	NA
Woeltje et al. [25]	1 hospital	4 non-ICU	US, Missouri	July 1st, 2005 to December 31, 2006	Fully automated	331 patients (391 positive blood culture)	CVC	CLABSI	NA	NA
Snyders et al. [26]	11 hospitals	17 ICU	US, Missouri	January 1st to June 30, 2011	Fully automated	518 patients (643 positive blood culture)	CVC	CLABSI	NA	NA, but adult patients
Bouam et al. [20]	1 Hospital	7 high-risk units	France, Créteil	11-week period	Fully automated	548 blood cultures	Unspecified	CRBSI	NA	NA
Hota et al. [24]	4 Hospitals	NA	US, Chicago	NA	Fully automated	NA	CVC	CLABSI	NA	NA
Lin et al. [27]	4 Medical centers	8 medical and surgical ICUs	US, Chicago, Columbus, St. Louis	January 1, 2004- June 30, 2007	Fully automated	1251 blood culture episodes, a random sample of 664 episodes was selected	CVC	CLABSI	NA	NA
Kaiser et al. [23]	1 Hospital	1 mixed ICU	Netherlands, Amsterdam	October 2009-October 2010	Semi automated	553 patients	CVC	CLABSI	6793 patient days of stay- 13.887 central line days	NA

ICU: intensive care unit; NA: not available; CVC: central venous catheter; CLABSI: Central Line associated bloodstream infections; CRBSI: Catheter related bloodstream infection; US: United States

### Definitions used to develop algorithms

Seven out of nine studies used CLABSIs and only two studies used CRBSIs as outcome. All studies adapted the CDC NHSN definitions to develop automated algorithms. It is noteworthy that the NHSN CLABSI definition has been modified over the years. One of the major changes since 2008 was the removal of the criterion considering a single positive blood culture with a common skin contaminant (CSC) as a CLABSI in the presence of relevant clinical symptoms and antimicrobials. Three studies used the pre-2008 definition and the four others used the post-2008 definition. Two studies used CRBSI as the outcome [19, 20]. One of the studies defined CRBSI with a positive quantitative tip culture growing ≥ 10^3^ CFU/mL with the presence of local signs of infection at the catheter insertion site and systemic signs of infection (such as fever or increased white blood cell count), or both [20]. Bellini et al. defined CRBSIs as simultaneous isolation of the same organism from blood and catheter tip culture (> 15 CFU/catheter tip) [19]. Clinical or therapeutic criteria were not included in these two CRBSI automated systems, because they were not available in their local hospital’s information system.

### Dataset used for indicators and denominators

Table 2 describes the type of data used in each study. All studies used administrative data in their automated algorithms, including admission dates (all studies) and discharge dates [21–24]. Microbiological data were automatically extracted in eight out of the nine studies [19–22, 24–27], with dates of blood culture sampling in fours studies [20, 22, 25, 26]. Only one study used a semi-automated CLABSI surveillance system with manual extraction of microbiological results [23]. The two studies evaluating an automated CRBSI detection system used blood and catheter tip culture results. Microorganisms were considered identical if the species identification and antimicrobial susceptibility profile matched [19, 20]. Snyders et al. evaluated the addition of cultures from other body sites (a sterile site, skin, wound, respiratory tract) in the algorithm [26]. Hota et al. included all positive and negative cultures from blood and other body sites, except catheter tips and surveillance cultures [24]. Lin et al. included wound or any non-blood cultures to identify primary BSI [27].

**Table 2:** Type of data automatically extracted and integrated in the different algorithms

Study	Clinical data	Laboratory (microbiological data)	Administrative data	CVC use	Antimicrobial data
Snyders et al. [26]	Temperature (> 38.0)	Positive blood culture, positive culture from other body sites (a sterile site, skin, wound, respiratory tract), culture collection date	Date of admission	Presence of central line	None
Kaiser et al. [23]	Temperature (T max > 38.0) and blood pressure (systolic < 90)	Not automated (manually extracted)	Date of admission and discharge	Presence of following central lines: Arterial, dialysis, number of lumens	Administration of specific antimicrobials: vancomycin, flucloxacillin, ceftriaxone, ciprofloxacin, imipenem, fluconazole, voriconazole
Bouam et al. [20]	None	Positive blood cultures with antimicrobial susceptibility testing results; positive quantitative catheter tip culture results; bacteriology sampling date	Date of admission, date of unit transfers,	None	None
Trick et al. [21]	None	Positive blood culture	Date of admission and discharge	Manual determination of the presence of a CVC	Vancomycin administration date
Lin et al. [27]	NA	Positive blood culture, positive wound culture, positive non-blood culture	Date of admission	Presence of central line	None
Woeltje et al. [22]	Temperature (> 38.0)	Positive blood culture and sampling date	Date of admission, discharge	Presence of central line	Inpatient medication orders: treatment with vancomycin
Woeltje et al. [25]	Temperature (> 38.0)	Positive blood culture and sampling date	Date of admission	Presence of central line	None
Bellini et al. [19]	None	Positive blood and catheter culture, date, species identification and antimicrobial susceptibility profile	Patient ID, ward, date of admission	None	None
Hota et al. [24]	None	Positive and negative culture from blood and other body sites, but excluding catheter tips or surveillance cultures	Admission, discharge and transfer date	None	Pharmacy dispensing or ordering data, to assess if vancomycin prescriptions had occurred

NA: not available; max: maximal; patient ID: patient identification; CVC: central venous catheter

The presence of a central line was included in the dataset of five studies [22, 23, 25–27]. Four studies included clinical data, such as fever (> 38.0 °C) [22, 23, 25, 26] or blood pressure [23], in their automated system. Only one study used machine learning to convert free text data into structured data to be used for automated surveillance [24]. Four studies included antimicrobial therapy data in their automated system, and all of these studies used vancomycin prescription data as an indicator for CLABSIs [21–24]. Kaiser et al. added other relevant antimicrobials to their automated system [23]. Six studies provided details of the denominator used, five of which chose central line-days (e.g., CLABSI rate per 1000 central line-days) and one study used patient-days (CLABSI rate per 1000 patient-days) as the denominator.

### Algorithms

Four studies reported a single algorithm [20, 23, 24, 27], while five studies evaluated more than one combination of algorithm rules. All studies compared the performance of the automated algorithms with the reference standard, defined as manual chart review. The most common rules used in the different algorithms were categorized as hospital-acquired rules, infection rules (infection *versus* contamination), deduplication, secondary BSI rules (secondary *versus* primary BSI), and catheter-related rules (Table 3). Most of the studies defined a hospital-acquisition rule, as blood culture collected more than 48 h after hospital admission [20, 22, 24–26].The majority of the studies defined infection rule as: ≥1 blood culture with non-CSC organism(s), ≥ 2 blood cultures with CSC organisms (same species), and ≥ 1 of the following signs or symptoms: fever (> 38.0 °C), chills, or hypotension. Three studies defined the secondary BSI rule as positive culture of the same organism identified in blood from another body site [20, 22, 25]. Different deduplication and catheter associated rules were applied in the included studies.

**Table 3:** Rules categories and most common rules applied

Rule category	Most common algorithm rules	Studies in which the algorithm rule is used
Hospital acquisition	Blood culture collected > 48 h after hospital admission	Woeltje et al. [22], Woeltje et al. [25], Hota et al. [24], Snyders et al. [26], Bouam et al. [20]
	Blood culture collected ≥ 3 days after hospital admission	Trick et al. [21], Bellini et al. [19], Lin et al. [27]
Infection	≥ 1 blood culture with non-CSC organism(s)	Lin et al. [27], Trick et al. [21], Woeltje et al. [25], Woeltje et al. [22], Hota et al. [24], Bellini et al. [19], Bouam et al. [20]
	≥ 1 blood culture with CSC organisms and appropriate antimicrobial therapy	Trick et al. [21], Hota et al. [24], Woeltje et al. [22], Bouam et al. [20]
	≥ 2 blood cultures with CSC organisms (same species)	Lin et al. [27], Trick et al. [21], Hota et al. [24], Woeltje et al. [25], Woeltje et al. [22], Snyders et al. [26], Bouam et al. [20]
	≥ 1 of the following signs or symptoms: fever (> 38.0 °C), chills, or hypotension	Woeltje et al. [22], Woeltje et al. [25], Snyders et al. [26], Bouam et al. [20], Kaiser et al. [23]
Duplication	Blood cultures yield the same organism within 7 h are considered as duplicates	Bellini et al. [19]
	Keep only first unique isolate scored as an infection within a 30-day period	Lin et al. [27], Hota et al. [24], Trick et al. [21]
	Any other positive blood culture within 7 days of the CLABSI culture	Snyders et al. [26], Woeltje et al. [25]
	Any positive culture with the same organism as the first positive CLABSI culture within the 14 days of the initial culture	Snyders et al. [26], Woeltje et al. [25]
	Any positive culture with the same organism as the CLABSI culture within 7 days of the initial culture	Woeltje et al. [22]
Secondary BSI	Positive culture of the same organism identified in blood from another body site	Woeltje et al. [25], Woeltje et al. [22], Bouam et al. [20]
	Identical organisms seen in both a non-blood specimen and a blood culture, where the non-blood specimen was collected − 21 days and + 7 days after the blood culture	Snyders et al. [26]
	Organism recovered from blood, also recovered from a non-blood culture: 3–7 days after the blood culture or during the entire length of stay	Trick et al. [21]
	Identical CSC species that are isolated from wound culture during day − 3 to + 7 of positive blood isolate or entire admission	Lin et al. [27], Hota et al. [24]
	Identical Non-CSC species that are isolated from any non-blood culture during day − 3 to + 7 of positive blood isolate or entire admission	Lin et al. [27], Hota et al. [24]
Catheter associated/related	Central line present ≤ 48 h before collection of culture samples	Woeltje et al. [22], Lin et al. [27]
	Central venous catheter in situ at the time of positive blood culture or discontinued within 48 h before positive blood culture	Woeltje et al. [25], Snyders et al. [26]
	Same organism cultured from a catheter tip at the time of positive culture ± 72 h interval	Bellini et al. [19]
	Patient with a positive quantitative tip culture growing at least 10^3^ CFU/mL	Bouam et al. [20]

*Abbreviations* CSC: common skin contaminant; CFU: colony forming unit; CLABSI: Central Line associated bloodstream infections; BSI: Bloodstream Infection

## Discussion

### Summary of evidence

Our scoping review identified nine studies from 2000 to 2021 on automated CLABSI/CRBSI surveillance. The automated surveillance systems were heterogeneous in terms of the definitions of CLABSI and CRBSI, the datasets and denominators used, and the combination of rules in each algorithm. This highlights the necessity for more research and alignment regarding definitions suitable for large-scale automated surveillance, appropriate data sources, methods for estimating denominators, and the development and implementation of algorithms across various contexts.

Overall, CLABSI was the most frequent outcome used in the included studies. Interestingly, long-term, midlines and PICC lines were disregarded in automated surveillance systems. CRBSI definition requires catheter removal and catheter tip culture. Therefore, CRBSI surveillance allows a higher degree of certainty in attributing the catheter as the source of the BSI [28], compared to the conventional CLABSI definition. However, catheter tip cultures are not commonly performed in many countries [29]. In spite of the fact that peripheral intravenous catheter associated BSI is a rare event, the frequent use of these catheters in healthcare makes this outcome relevant. In the majority of the studies, the adoption of fully automated systems required adjustments to the surveillance definitions, potentially resulting in information loss. Furthermore, over the years, distinct definitions for CLABSI and CRBSI surveillance have been changed, making it challenging to compare the results generated by the various automated systems. For instance, Lin et al. modified their categorization of blood culture episodes during their study due to the removal of the NHSN CLABSI criterion that classified a single positive blood culture with a CSC as a CLABSI in the presence of clinical symptoms [27]. Fully automated systems generally require adaptation of definitions used in the context of manual surveillance, which could lead to loss of clinical relevance. Consensus should be reached on surveillance definitions that are suitable for automated surveillance, and based in priority on data that are readily available in the electronic health records.

All studies used algorithms that combined multiple data sources to identify CLABSI/CRBSI. The majority of studies relied on admission data and microbiology culture results, which are usually in a structured format. Many of the studies included the presence of IVC in their automated system. Only few studies included antimicrobial use and clinical data such as vital signs. Bouam et al. presumed that the lower sensitivity of their automated system could be explained with the lack of automated clinical signs and symptoms [20]. When developing an automated surveillance, a minimal dataset that include the most important variables of interest should be developed, and it should carefully include details of the data sources. The minimal dataset should allow extraction of data from electronic health records [14]. Higher specificity of these automated surveillance systems could be achieved in the future by better capturing the data included in the electronic health records using more advanced IT process, such as text mining [14].

When computing rates of CLABSI, catheter-days is calculated as a denominator. Patient-days allows to calculate CVC utilization [11, 25, 30]. Four studies calculated their denominator as catheter-days [22, 23, 25, 27]. Automation of denominator calculation for CLABSI has not been explained in some of the included studies, as device use was not documented in a structured format. None of the studies discussed denominator calculations for CRBSI. To correctly measure incidence densities, we recommend both patient-days and catheter-days data to be extracted and used as denominator.

Based on the surveillance definitions recommended during the time of each study, different algorithms have been developed and assessed on various study populations. We categorized the most frequent rules and steps used in the algorithms as hospital-acquired rule, infection rule (infection *versus* contamination), deduplication, secondary BSI rule (secondary *versus* primary BSI), and catheter-associated rule. Most of the studies reported an *overestimation* of CLABSI/CRBSI with automated surveillance compared with manual surveillance. Bellini et al. observed that prolonged BSI episodes such as *Candida sp*. fungemia could be counted multiple times; therefore, these investigators improved their algorithm performance by considering only one episode of *Candida sp*. fungemia for the entire hospital stay [19]. Woeltje et al. observed that some infections classified as CLABSI by the automated system were secondary infections with a site culture that was not included in the automated algorithms [25]. The differential time to positivity, which is an important criterion to identify CRBSI, was not included in any of the algorithms. Translating manual surveillance definitions into automated rules may lead to a great variety of algorithms, and therefore different CLABSI rates were produced by these algorithms. As part of a standardization initiative, it is essential to create guidelines and establish a standardized approach in order to provide valid results. Additionally, a comprehensive report detailing the application of these rules and the combinations of rules applied to the data should be provided [18].

### Limitations

Our scoping review has limitations. First, the aim of the scoping review was to provide a broad overview of the literature, rather than a detailed synthesis of the outcomes. Therefore, our study group conducted a separate analysis to assess the performance of these algorithms [18]. Second, we only included peer-reviewed literature, including grey literature may have identified additional automated systems that have been implemented and evaluated, especially in commercially available packages. Third, we performed a scoping review and a critical appraisal of the included study was not performed. Finally, since we restricted our review to studies published in English, we might have missed relevant work published in other languages.

## Conclusions

This scoping review sought to examine the current literature on the development of automated systems to monitor CLABSI/CRBSI through a systematic search of the literature. The findings suggest that, while efforts to shift from traditional to automated CLABSI/CRBSI surveillance have been made over the past two decades, a need for further research is required to optimize these automated surveillance methods. More extensive guidelines and studies are needed to create and deploy algorithms for CLABSI/CRBSI detection, with standardized definitions, appropriate data sources, and denominator calculations. Moreover, providing in-depth insights into the design of automated systems will help in continuously enhancing algorithm performance, thereby facilitating the widespread implementation of automated systems across diverse healthcare settings.

### Electronic supplementary material

Below is the link to the electronic supplementary material.

Supplementary Material 1: Search strategy.

## Data Availability

No datasets were generated or analysed during the current study.
