# Supplementary material for: Systematic scoping review of automated systems for the surveillance of healthcare-associated bloodstream infections related to intravascular catheters
Source: Antimicrob Resist Infect Control. 2024 Feb 28;13:25. doi: 10.1186/s13756-024-01380-x (PMC10903068; doi:10.1186/s13756-024-01380-x)
Supplement: Supplementary file 1 — Supplementary Material 1: Search strategy. [file 13756_2024_1380_MOESM1_ESM.docx]

**Supplementary materials**

**Search strategy**

**Key-words algorithm used in PubMed** (https://pubmed.ncbi.nlm.nih.gov/)

((((catheter[Title/Abstract]) AND ((infect*[Title/Abstract]) OR (bact*[Title/Abstract]))) AND (((automat*[Title/Abstract]) OR (electronic*[Title/Abstract])) OR (comput*[Title/Abstract]))) AND ((surveil*[Title/Abstract]) OR (monitor*[Title/Abstract])) AND (2000/1/1:2021/12/31[pdat]))

OR

((((hospital acquir*[Title/Abstract]) AND ((infect*[Title/Abstract]) OR (bact*[Title/Abstract]))) AND (((automat*[Title/Abstract]) OR (electronic*[Title/Abstract])) OR (comput*[Title/Abstract]))) AND ((surveil*[Title/Abstract]) OR (monitor*[Title/Abstract])) AND (2000/1/1:2021/12/31[pdat]))

**Key-words algorithm used in EMBASE** (https://www.embase.com/)

catheter:ab,ti AND ('infect*':ab,ti OR 'bact*':ab,ti) AND ('automat*':ab,ti OR 'electronic*':ab,ti OR 'comput*':ab,ti) AND ('surveil*':ab,ti OR 'monitor*':ab,ti) AND [1-1-2000]/sd NOT [1-1-2022]/sd

OR

'hospital acquir*':ab,ti AND ('infect*':ab,ti OR 'bact*':ab,ti) AND ('automat*':ab,ti OR 'electronic*':ab,ti OR 'comput*':ab,ti) AND ('surveil*':ab,ti OR 'monitor*':ab,ti) AND [1-1-2000]/sd NOT [1-1-2022]/sd
